# Guy's Hospital Reports

**Published:** 1842-07

**Authors:** 


### Art. X.

*Guy's Hospital Reports.* No. XIII. Oct. 1841.—*London,* 8vo.

The Number before us fully sustains the high character which these valuable reports have maintained from their commencement. We present our readers with the following abstract of its contents.

i. The first paper is part of a communication by Dr. G. H. Barlow, *On certain Diseases originating in early Youth.* We shall defer any notice of it until its completion.

ii. *Medico-legal Report of a Case of Poisoning with Arsenic,* by Mr. Taylor. This is another valuable contribution to forensic medicine, from a gentleman to whom the profession is already much indebted. We shall endeavour to give as complete an abstract as we are able of its leading features, earnestly recommending our readers to peruse the whole.

The case occurred near Wallingford, in Berkshire. The unfortunate individual was a married man, aged 28, who lived on bad terms with his wife, on account of an improper intimacy which existed between her and another man. He was seen by Mr. Breach, a surgeon, on January 27, 1841, apparently, then, labouring under common diarrhœa, which was very prevalent in the neighbourhood, and with no evident symptoms of danger. Compound chalk powder with opium was prescribed. He died next day; and suspicions being excited, a coroner's inquest was held, and the body examined. The dissection was made fifty-nine hours after death. The body presented no unusual external appearances. The omentum was found highly congested and inflamed. The small intestines were of a vermilion-red colour throughout (internally ?), but diminishing in intensity from the duodenum downwards. There was no appearance of ulceration in the intestinal canal, nor was any part of the peritoneum inflamed, (we presume the *attached* portions of the peritoneum are meant, for it is stated a few lines above, that the omentum was highly inflamed.) There was no effusion of lymph, and no adhesions could be detected. The stomach was removed, and shown to Mr. Taylor five days after the examination, having been kept, with its contents, in a sealed bottle during the interval. The whole of the mucous membrane was found to be of a dull red colour, the dulness being particularly well marked in the interspace between the cardia and pylorus. In the lesser curvature, between these openings, there were three rather elevated lines, consisting of firmly adherent mucus, intermixed with a white substance, having a streaky character, like cream, poured on a dark surface. These lines had a border of deep redness, which became gradually shaded off into the surrounding mucous membrane. On removing the mucus with a spatula, the stomach beneath presented a deep cinnabar-red colour. There were several small patches of ulceration about this part of the viscus; and near the cardiac orifice were two ulcers, almost circular, and each about the size of a sixpence. When first examined they were covered by a white powder, and surrounded by dark effused blood. The rugæ were numerous and highly inflamed at the margins. When first seen, a white pasty matter was diffused in patches over the whole surface. The coats of the organ were not altered in thickness or consistence.

We must pass over the details of the analysis, which was conducted with great care and minuteness. No arsenic whatever was discovered in the fluid contents, but it was most unequivocally detected in the white matter and mucus adhering to the coats of the stomach, by all the tests upon which reliance can be placed. But though we have not room for a full account of the various processes, there are two points connected with them, which cannot with propriety be omitted, because they are of much importance in themselves, and are not yet generally known to the profession.

The first is a simple method, lately recommended by Mr. Marsh, for distinguishing the arsenical from the antimonial stain in his apparatus:

"Place a few drops of ammonio-nitrate of silver on a plate of glass, or in a small saucer, and hold it over the flame, at about half an inch from the point. If arsenic be present, yellow arsenite of silver will be formed; if antimony, according to Mr. Marsh, a white precipitate results: in the latter case, however, I have found the liquid blackened, from the reduction of the silver. I have since tried this with seleniuretted hydrogen, and found the liquid blackened: when the hydrogen is free from any foreign impregnation, the silver is also reduced. Hence, in the effect on the ammonio-nitrate of silver thus applied, we have a strong proof of the presence or absence of arsenic." (p. 271.)

With reference to the employment of Marsh's apparatus, it must never be forgotten that many specimens of sulph. acid, as sold in the shops, are contaminated with arsenic and selenium, (the latter of which gives a similar stain to the former,) and that frequent trials of the apparatus, *before introducing the suspected liquid*, are therefore absolutely essential.

The second point to which we would advert, is a modification of the copper test, which is not commonly noticed by medico-legal writers, but which, in Mr. Taylor's opinion, may be safely resorted to by practitioners.

"Having thrown down so much of the green precipitate as we possibly can, by adding the copper test to the suspected liquid, we wash, collect, and dry it. A small quantity of it, finely powdered, is then to be introduced into a minute tube, and very gently heated over the flame of a spirit-lamp. When this experiment is carefully performed a ring of small, octohedral crystals will appear, if the salt be arsenite of copper. These become sublimed and fixed on the glass tube, a little above the powder. These crystals of arsenic are plainly perceptible to the eye, from their highly refracting properties; and their triangular surfaces are easily distinguished by a lens of low power. If the heat be rapidly applied, there is produced only a dense, white crust of arsenic, in an uncrystalline state. The application of the copper test is, it appears to me, useless without this corroborative experiment. This is by far the best criterion of the nature of the precipitate; and is much more satisfactory than the plan commonly recommended, of distinguishing the arsenite of copper from other salts of that metal, by its solubility or insolubility in certain menstrua. To the medical jurist the result is interesting; since it is a most satisfactory method of reproducing the poison in the state in which it was actually taken. Arsenite of copper may be reduced by charcoal, (not by black flux;) but the result is never so satisfactory as the production of the crystals of arsenious acid, according to the method above mentioned." (p. 272.)

Proceeding now to the consideration of this case, as one involving a criminal accusation, we meet with several points of much importance, and no small interest to a medical man. The wife of the deceased and her paramour were both committed on a charge of murder, but as there was no direct evidence against the latter, he was discharged by the grand jury, and the female prisoner took her trial alone. Mr. Taylor and Mr. Breach were summoned on the part of the crown, to give evidence of the death by poisoning. It is of great importance that any one who is liable to be cited as a medical witness upon an occasion like the present, should be well prepared for the kind of questions he is likely to be asked, and we shall therefore shortly mention those that were put by counsel on this trial. Mr. Breach was asked, whether the diarrhœa might not have caused the man's death, but we need scarcely say that his opinion was conclusive on this point. He was then asked, whether arsenic might not have been accidentally mixed up with the powders sent by him to the deceased, and whether he kept arsenic among his medicines. The answers were, That he prepared the powders himself—that he had prescribed similar ones on the same day for other patients, without bad results—and that the only arsenical preparation he kept was a solution. He was next required to describe the method of analysis employed—to account for the safe custody of the stomach and its contents, until the analysis was made, and also of the tubes and plates containing the reduced metal. He was then asked the probable quantity of reduced metal in each tube, and whether it would be possible to convert all the poison, diffused through the stomach and intestines, to the state of metal.

The questions put to Mr. Taylor were, chiefly, Whether ulceration of the mucous membrane of the stomach was a common effect of arsenical poisoning—what was the earliest period at which it might take place—the earliest period for the occurrence of severe inflammation of the mucous membrane—the quantity of arsenic required to kill an adult—how much was found in deceased's stomach, and whether this was sufficient to destroy life? Again: How many tests were employed—which was the most satisfactory—and why, if the test of reduction were satisfactory, were others afterwards employed? "This last," says Mr. Taylor, "was an ingenious question; as, by the application of so many tests, it would seem as if we doubted the correctness of the inference drawn from those first employed. I answered it by saying that the various tests were employed, not merely to corroborate each other, but for the acquisition of experience, on their general efficacy and relative value."

In reference to the question of the earliest period at which severe inflammation of the mucous membrane could occur, Mr. Taylor observes:

"This is a question of some importance, although it does not seem to have attracted the notice of medico-legal writers. The earliest period at which I have heard of inflammation of the stomach being found, was about six or eight hours after the poison had been taken. A very important addition to our knowledge on this subject has been lately made by Mr. Foster, of Huntingdon. He has communicated three cases of poisoning by arsenic, to a medical periodical. A woman took arsenic, and administered the same poison to two of her children, the one aged two years and half, and the other an infant of five months. The mother died in three hours and half, the elder child in less than two hours, and the infant in six hours and half after having taken the poison. The quantity of arsenic swallowed could not be ascertained. The mucous membrane of the stomach, in each case, was found highly inflamed, but less so in the case of the mother than in the children. There was no ulceration. By these cases two new facts are added to our knowledge of arsenical poisoning: 1, that this poison may destroy life within two hours; and, 2, that in that short period acute inflammation of the stomach may be perfectly established." (p. 278.)

Mr. Taylor's analysis of the evidence adduced upon the trial is extremely interesting, and will amply repay the trouble of a careful perusal; we can give but a very short sketch. It was proved that the prisoner, three days before the death of her husband, had secretly applied to her sister to procure for her some arsenic, for the alleged purpose of destroying mice, and that two packages, containing about ʒj. of arsenic, were delivered into her hands about twelve o'clock of the day on which he died. These packages were not forthcoming after his death, and the prisoner affirmed that she had directed a woman, who was sweeping up the house, to throw them into the fire. It was also proved, that on the morning of the decease, before the arrival of the poison, the prisoner told one of the witnesses who went to the house, that her husband was "something better," that about midday he became violently ill, and that he died about five minutes after eleven, p. m. having, by her own admission, suffered severely from pain and vomiting. (It should be observed that all the vomited matters had been thrown away.) No medical assistance was applied for during the time of his severe illness, and when Mr. Breach told her that an examination was requisite, she became agitated, and said "What do the people expect to find? I am sure he came by a natural death. However, *I* gave him nothing!"

We believe there are few who could read even this slight abstract, and still fewer who could peruse the detailed account given by Mr. Taylor, without feeling at once convinced that the wife was guilty of the charge laid against her. The unfortunate man was seen by no one, except the prisoner, until he was evidently labouring under the effects of the poison, and there was not the remotest shadow of proof that he had either taken it himself or had it accidentally administered to him: nevertheless, the judge stopped the proceedings, and directed the jury to discharge the prisoner, on the grounds, "that this case was one of suspicion only—that there was no proof of any motive, on the part of the prisoner, for committing the act—nor was there any *direct* evidence of her having administered the poison to the deceased." The alleged absence of motive appears to us a very singular reason; we should have thought the adulterous intercourse, which was known to have existed between the prisoner and another man, was quite sufficient. There was probably no *direct* evidence of her having given the poison; but in how few cases can this be proved? Very little precaution is required to conceal the act so far, and in this instance it is perfectly clear that, either the man committed suicide or she was the murderer. Upon the whole, we cannot avoid the conclusion, that the ends of justice would have been better served, by allowing the trial to take its fair course, instead of smothering it in such an off-hand manner as was done on this occasion.

III. *A Case of Abdominal Effusion, resulting from Mesenteric Tumour, by* Dr. H. Marshall Hughes; *with Observations on the Effused Fluid, by* Dr. G. O. Rees. The patient, a young man aged twenty, by occupation a wire-weaver, had been particularly robust and free from ailment until about a year before he came under Dr. Hughes's care. In childhood he had never had any indication of mesenteric affection, or other abdominal disease. When first seen, (Dec. 31, 1840,) he appeared to be suffering from functional disorder of the stomach, which was stated to have been his chief ailment for some months previous, and he was treated accordingly, up to the 26th of January; when, upon being visited at home, he was found labouring under the same symptoms as before, with the addition of considerable tension of the abdomen, arising chiefly from flatulence. No tumour was detected, but there was some indistinct fluctuation. From this date he became progressively worse; the distension of the abdomen increasing with great rapidity, and the body becoming most singularly emaciated. No means of treatment were of any avail, and he died on the 17th of February, "being," says Dr. Hughes, "more, and especially more quickly, emaciated than any individual I had before seen." The body was examined twenty-two hours after death.

"Not a particle of fat was to be seen, either in the integuments or omentum. The peritoneal cavity contained from seven to eight quarts of rather thick and perfectly milky fluid, aptly compared, from its appearance, to almond emulsion. The peritoneum was not vascular, excepting over a depending portion of the ileum, but was universally sprinkled with minute white specks; by far the larger portion of which was easily removed by gentle friction, and consisted of delicate and almost capillary shreds, evidently deposited from the milky fluid, but some of which were as clearly firmly adherent to, if not produced by, the membrane itself. They were translucent, angular, and elongated, rather than rounded; and, in external appearance, bore a much more striking resemblance to the ova of *pediculi capitis* than to any form of tubercles. In the centre of the abdomen, resting on the spine, was a rounded nodulated tumour, as large as a twopenny loaf, which consisted of several agglomerated mesenteric glands; some of which were as large as a small orange, and, when divided, presented a soft, pinkish, pultaceous mass; from which, upon very slight pressure, exuded a white, cream-like fluid, which appeared to constitute a part of the deposit itself; others were of a dull, white colour, drier, and more granular, the whole exhibiting, both exteriorly and upon the exposed section, the general characters of cerebriform cancer. Other glands of the mesentery were more or less enlarged and opaque, some equalling the size of marbles and of pigeon's eggs. Some of the inguinal glands were also considerably enlarged, but contained no heterologue [gous] deposit. Several convolutions of the intestines, and the transverse arch of the colon were adherent to the tumour. They all, however, appeared healthy except the colon, which in two places was contracted and puckered round two white spots as large as a shilling, which were white, firm, and semi-cartilaginous in appearance. Opposite to these spots, the mucous membrane was entirely wanting; and their cut surface presented the same physical characters as the early stage of scirrhous pylorus. One tubercular-looking body *only*, about the size of a pea, was discovered in the mesentery, close to a fold of the ileum. The liver, spleen, and kidneys were healthy. The pancreas was, unfortunately, not examined. Numerous lacteals—large tortuous, varicose, and distended, some with a milky and others with a clear fluid—were observed in almost all parts of the mesentery; but, in consequence of the sections already made, and of the examination occurring in a private house, no attempt at injection, for the purpose of discovering any lesion, could be made with any probable chance of success." (p. 300.)

Some of the fluid was sent to Dr. Rees for examination, who found it to contain *chyle* in considerable quantity; how much he could not determine. When agitated with æther the fluid separated into three parts, of which the upper was a solution of fatty matter, the lower serum, and the middle a floating mass of chylous matter.

This is assuredly a very singular case, but Dr. Hughes is mistaken in supposing it to be unique—the effusion of milky or chylous fluid into the cavity of the abdomen has been noticed by several pathologists, and ascribed to rupture of a lymphatic vessel. (Vide Copland's Dict., art. Dropsy, p. 605.)

iv. *On Acute Aortitis, by* Dr. Norman Chevers. This is a valuable paper, comprising, as the author informs us, some of the facts elicited in the careful examination of nearly 400 aortæ, from patients in Guy's Hospital, in a great proportion of whose cases he had an opportunity of watching the symptoms during life.

Dr. Chevers is of opinion that the arterial tissues are rarely if ever subjected to chronic inflammation, precisely analogous to what occurs in other organs, and he therefore limits the forms under which the inflammatory state developes itself in these textures, to those which are usually distinguished by the terms acute and sub-acute. Our readers will judge for themselves how far the facts related bear out this doctrine.

Acute inflammation of the thoracic aorta appears to occur, in nearly all cases, either as a direct result or as a complication of some other morbid process existing in the system. In the early states very few of the implicated structures become the seats of morbid depositions; but as the diseased action advances, certain effusions take place, the most yielding tissues suffering first. Thus, when the aorta has been long subjected to irregular distension and irritation, in consequence of hypertrophy of the left ventricle, the outer cellular portion of its sheath is often found œdematous and spongy.

We doubt, however, whether this can be regarded as a mark of inflammatory action; it is probably the result of obstructed circulation through the coats of the vessel. Dr. Chivers has also noticed the existence of numerous spots of red or purple ecchymosis in this coat, varying from one to four lines in diameter, occupying the cells immediately in contact with the denser portion of the external coat, and remaining attached to it after the loose filamentary tissue of its sheath has been torn away. Numerous vessels can always be traced to the circumference of these spots, and they generally appear to have been so recently deposited, as to lead to the surmise that they are formed either shortly before death or during the last agony. These two forms of lesion have usually been observed in cases where the death-struggle has been protracted, as in hydrophobia, or accompanied with much suffocative action, as in cases of obstruction of the air-passages. They are also frequent in extensive thoracic inflammations, especially asthenic pleuritis. Dr. Chevers has never discovered such appearances in the aorta below the diaphragm, nor in any of the smaller arterial trunks. It does not appear that the contractile coat of the aorta undergoes any change in the early stages of inflammation beyond that of softening, rendering it liable to transverse rupture at its upper part. Dr. Chevers believes such an event is more generally attributable to acute than to chronic change. Of the correctness of this opinion we entertain very great doubts; we believe that, in the majority of instances in which this accident has occurred, the coats of the vessel have exhibited decided marks of slow and continued diseased action.

The lining membrane of the aorta and the structures immediately subsidiary to it, present two distinct forms of alteration when acutely inflamed. We shall shortly notice both.

*First Type.* This appears to result from a very active form of acute, adhesive inflammation; a single layer of coagulated lymph, of variable thickness, being effused over a greater or less surface of the serous membrane. This kind of effusion is, however, rarely seen in its original condition, unless death ensues very rapidly, for it is soon covered by masses of coagulum from the blood. It is occasionally noticed in over-worked horses which have died from thoracic inflammation, and Dr. Chivers has observed it in one case in the human subject. A similar condition exists in the smaller vessels in dry gangrene; the previously diseased walls becoming acutely affected and, as a consequence, covered with plastic lymph, upon which clots are deposited and organized. There is this difference between the two cases, that, while the whole circumference of the smaller vessels is occupied by the effusion, it is generally confined to one side of the caliber of the aorta, and yet the vessel may be materially obstructed, or even entirely closed, by the large size of the organizable clots found upon it. There is a preparation in the museum of Guy's Hospital which well illustrates this point: the aorta is rendered completely impervious, from the origin of the inferior mesentric artery downwards, by a firm mass of pale fibrin. The aortic valves bore traces of acute inflammation, and the lower part of the vessels had undergone extensive ossific changes. It does not appear that a scabrous state of the walls of an artery renders it liable to the formation of these clots, unless an acute attack supervene; for ossified vessels often remain unobstructed for years.

This form of disease may occur in one of two states of the circulation, or from a combination of both. It may arise while the flow of blood through the vessel is feeble or unnaturally slow, from diminished power of the heart. Thus, in some cases of exhaustion during the latter stages of adynamic disease, inflammation of a low type is set up in the aorta, and lymph being effused from its lining, the current of blood has not strength to prevent the formation of a false membrane, nor to wash it off when produced. Or it may occur from some actual impediment producing a degree of stasis in the current. In certain stages of Bright's disease there is a great tendency to these inflammatory effusions, and the obstructed circulation through the kidneys, liver, and spleen, combined with the languor of the heart's action, gives rise to the formation of the blood-deposits.

*Second Type.* This bears a close resemblance to the worst forms of erysipelas, in the rapidity of its course, the character of its products, and its selection of the most debilitated and cachectic individuals as the subjects of its ravages. In a man who died a few days after the operation of lithotomy, the aorta presented the following appearances:

"The outer coat was traversed by a multitude of enlarged vessels, many of which would have admitted a bristle, inosculating with each other by minute branches. A few small, light-red ecchymoses adhered to the denser portion of the outer coat. The lining membrane was in some places of a dark cornelian hue; in others it presented a paler shade of red; and in one spot, extending near the origins of the intercostal arteries, this had deepened to a blackish violet tint. The redness was chiefly owing to the presence of what appeared to be a minute network of injected vessels, disposed nearly equally over the whole sub-serous tissue; the partial deepening of colour probably, in some degree, depended upon cadaveric change. The internal coat was neither thickened nor unusually lacerable: its redness extended slightly in some places to the middle tunic, which presented little change beyond an appearance of unusual yellowness." (p. 314.)

This form of aortitis only appears in the most enfeebled constitutions, either during the latter stages of ataxic fevers, or in the subjects of chronic visceral disease, when suffering under the effects of severe operations, profuse hemorrhage, phlebitis, or arteritis. The effused matter is not sufficiently plastic to resist the current of the circulation, and therefore neither lymph nor coagula form upon the interior of the vessel, and the morbid action continues to spread onwards, until nearly the whole of the great arteries are involved in the fatal mischief. It appears to be identical with the erysipelatous arteritis which was observed by Cline and Abernethy after operations for aneurism, and which also occasionally results from slight injuries of the extremities, or even idiopathically from purely constitutional causes.

Pathologists are not agreed as to the nature of the red stains so frequently presented by arterial linings, namely, whether they are results of cadaveric changes, or evidences of diseased action during life. Dr. Chevers has made some interesting experiments for the elucidation of this point, and also for the purpose of ascertaining in what degree various conditions of the internal membrane before death influence the production of stains afterwards.

"I placed," he says, "four portions of aortæ, from different subjects, in blood recently drawn, (having broken up the clot and added the serum;) and allowed them to macerate in it for twenty-four hours, the usual average time at which bodies are inspected after death. [The experiment was performed during the winter.] When removed from the blood, 1, a portion of pale and recent aorta was found to have undergone no change. 2. In another portion, which, when removed from the body, was slightly stained in numerous patches and stripes, these parts were found to be losing their red tinge, and assuming a blackish or chocolate-brown colour, the intervals remaining clear. 3. A piece of aorta, in which portions of the lining had been red, but were becoming black from putrefaction, was little changed. 4. This was also the case in another vessel, where the inner membrane presented coloured spots, probably of inflammatory origin.

"The arteries were then again allowed to remain immersed for twenty-four hours, and at the end of that period it was found that, 1. All the tissues of the healthy artery were stained of a dull flesh-colour. 2. The surface of the stained vessel offered a generally darker hue, but the brownish stains were less defined. 3. The decomposing vessel was nearly equally throughout of a dirty-gray colour. 4. The inflamed aorta had become little changed, except that its reddened spots were darker, and that the remainder of its interior had lost its polish.

"From this experiment I think we may deduce the following inferences: That, during cold and temperate weather, stains from imbibition will not be produced in tolerably healthy arteries until after the usual time at which post-mortem examinations are made. That stains, very different from those produced during life, result from the imbibition of blood similar to that usually found in the aorta. Again: That a colour produced by inflammatory action becomes somewhat changed after death, by the long application of fluid blood." (pp. 316-17.)

When inflammation has continued long in the aorta, the lining membrane is generally raised in many places by solid masses of lymph in the sub-serous cellular tissue: these generally peel off with the membrane, and, until cut into, appear like red vesicles rising behind it. Dr. Chevers is of opinion, that so long as these masses retain their transparency and florid colour, the disease may be looked upon as recent; for shortly after their deposition a slight cloud of white opacity begins to appear in the centre of each, and gradually advances until the whole becomes of an almost cartilaginous toughness, and then slowly undergoes the atheromatous or ossific change. In these opinions he is directly opposed to M. Bizot, who contends that the masses of lymph are deposited upon the free surface of the membrane, and never undergo the ossific alteration. We believe that Dr. Chevers's views are by far the most sound, but our space will not admit of a full development of the argument.

It is very common to find the lining membrane of the aorta partially reddened, in cases where inflammation cannot be regarded as the cause, and where the stains, from their position and other circumstances, manifestly do *not* result from contact with blood, or gravitation of the fluid contents of the *vasa vasorum*. This appearance is explained in the following manner by Dr. Chevers:

"I endeavoured to account for this kind of reddening by assuming that, during life, the inner surface of the aorta is of a pink colour, resembling that of mucous membranes. Now, it may be observed, that, in the moment of death, the aorta, contracting, loses its cylindrical form; and, becoming flattened, its opposite surfaces are placed for a time completely in apposition. During a perfect contraction of this kind, the capillaries supplying the lining membrane must become emptied of their contents, which pass into the outer vasa vasis, leaving to the inner part of the tube its usual blanched appearance. But should any circumstance, occurring during or even very shortly after the last agony, as a failure in the action of the organic nerves at any point, prevent the cylinder from exerting or maintaining its tonicity at that spot, the internal surface would there retain its original florid aspect, while the remainder became entirely deprived of colour." (p. 319.)

Ingenious as this explanation assuredly is, we very much doubt if it will stand the test of examination. Is it true that the aorta becomes flattened at the moment of death? We feel convinced that such is not the case. The tonicity of the middle coat of arteries undoubtedly gives rise to contraction, and the cavity of a vessel may even be obliterated in this manner; but what results? Why, that we have a round cord instead of a cylinder; not a flat riband, as Dr. Chevers imagines. Again; supposing the tonicity of any part to be destroyed, (and the *possibility* of such an occurrence might readily be disputed,) would not the stains occupy the whole circumference of the artery, rather than occur in "marginated streaks and patches?" And, lastly, where have we the shadow of a proof of the pink colour of the inner surface of the aorta? But, indeed, a process of argumentation, which is avowedly based upon a pure assumption, is scarcely worth the trouble of demolishing. We are sorry to meet with anything of the kind in so admirable a paper.

In reference to the *causes of aortitis*, Dr. Chevers advances little that is new. The disease is evidently most liable to occur in debilitated and cachectic constitutions, from whatever circumstance such a state may have originated. He is inclined to think, that the attacks are frequently produced either by imperfectly decarbonized blood, as in cases where there is much pulmonary affection, or by a too highly azotized condition of that fluid, as in Bright's disease.

The same may be said of his observations upon the *symptoms* and *treatment;* they differ in no important respect from the accounts given by other writers. We should notice, however, that Dr. Chevers is decidedly opposed to the vigorous antiphlogistic treatment recommended and adopted by some continental practitioners; and considering the extreme rarity of a sthenic form of aortitis, we think his remarks are judicious.

v. *Cases of Malignant Disease of the Lungs, by* Dr. Hughes. This is a paper also which deserves perusal: not on account of anything novel in the statements, but simply because it contains the record of four interesting cases and dissections. The first two of these are evidently instances of cerebriform disease of the lung; the nature of the others, and particularly of the last, is much more obscure; indeed we feel very great doubts as to the propriety of including *it* in the class of malignant affections. A mere abstract of a paper like this would be of little value; and as we have not room for its entire transcription, we must content ourselves with a very few observations.

According to Dr. Stokes, there are two forms of this complaint: in the first, the organ itself is transformed into a cancerous mass; in the second, a tumour is developed external to the lung, which it ultimately displaces. Dr. Hughes would add to these a third and, as he believes, much more common form, viz. the dispersion of a number of rounded masses, varying in size, colour, and consistence, through the greater part of one or, more frequently, of both lungs, while disease of a similar nature exists at the same time in other parts of the body. Only one, however, of the cases narrated in the communication before us is an example of this variety.

In reference to *diagnosis*, we are unfortunately but little assisted by the researches of Dr. Hughes. The number of cases that have been accurately observed is far too small to admit of any positive deductions. Dr. Hughes thinks that an appearance of the sputa resembling "red currant jelly and water," the existence of perfect dulness on percussion in some part of the chest, obstruction of the superficial veins of the affected side, shown either by enlargement of the vessels themselves, or by œdema, are the chief signs to be relied on. Of course the coexistence of malignant tumours in some other parts of the body will materially aid the formation of a correct opinion, and much may also be gained from the history of each case.

VI. *Report of Cases requiring capital Operations, by* Bransby B. Cooper, Esq. There is little to detain us in this paper. Five cases of operation for aneurism are reported. In these we shall only notice two points.

1. In tying the femoral artery, instead of drawing the saphenous nerve outward, and then passing the aneurismal needle between the artery and vein, Mr. Cooper adopted the following plan:

"Immediately on opening the sheath, he passed an aneurismal needle, unarmed, below the whole of its contents, from without to within, and to such a distance, that its curved extremity appeared close to and on a level with the upper and inner edge of the artery. There were two branches in this case, running along the upper surface of the artery. Having gently detached these, by means of the blunt end of a small probe, Mr. Cooper 'tilted' them over the exposed end of the aneurismal needle; on which, therefore, remained lying only the artery and vein. Between these Mr. Cooper now insinuated the end of the probe; and through the space thus made, passed the needle, having removed it cautiously from its former situation. In this situation the needle was armed by an assistant, and the ligature then secured in the usual way. This method appeared to place the separation of the artery from the nerves and vein more under the command of the operator, while it caused the least possible disturbance to its cellular attachments." (p. 354.)

With all due deference to Mr. Cooper, we must beg leave to differ essentially from him in our estimate of the value of this modification. In the first place, it appears sufficiently plain that it most unnecessarily detaches the vein from its sheath, which is avoided by the old method. Secondly, we cannot understand how the nerve is more likely to escape injury, seeing that the needle is withdrawn from its position after the nerve has been "tilted over" its extremity, and then requires to be inserted afresh. And, thirdly, we should conceive that, when the parts are deep-seated, it would be no easy matter to arm the needle after it has been passed round the vessel.

2. In applying a ligature to the common carotid, Mr. Cooper advises the first incision to be made nearer the mesial line than usual, in order that the sheath may be opened as much on the inner side as possible, by which means the difficulties often experienced from the overlapping of the jugular vein are avoided. If this method be adopted it will be found impossible to introduce the needle from without inwards, but it can be easily effected in the opposite direction. During the performance of the operation related, a circumstance of some physiological interest occurred. The patient made suddenly a peculiar barking noise, and he afterwards informed Mr. Cooper that it was quite involuntary, and appeared to be caused by something in the wound being pushed aside by the operator's fingers, and that it "was attended with a curious sensation, as of some one squeezing the lower part of his throat, and also a sudden weight at the stomach, and a disturbance in the bowels; so that he was, for a minute or two, afraid that he should not be able to retain his fæces." This was evidently caused by pressure upon the *par vagum.*

Two successful cases of *excision of the elbow-joint* are next related, but present no remarkable features. Mr. Cooper appears to regard anchylosis as the termination to be aimed at; but assuredly, under proper management, a much more favorable event may be reasonably expected. Many cases are on record in which the affected limb has regained powers of motion scarcely inferior to those of the healthy extremity.

The case of *steatomatous tumour* in the gluteal region, which concludes the paper, requires no particular notice.

vii. *On the Structure of the Blood-Corpuscle, by* Dr. G. O. Rees and Mr. S. Lane. According to the observations of these gentlemen, the corpuscle is a flattened cyst adherent at its centre, or axis, to a nucleus, and having a fluid surrounding that nucleus at all parts but those by which it adheres to the envelope. This structure is demonstrated in the following manner: a drop of blood, placed between glass and mica, being examined, and the ordinary appearances recognized, a drop of concentrated syrup was added, and the mixture submitted to the microscope. "The corpuscles had quite lost their original form, being collapsed and, in some instances, doubled on themselves; some of them had the appearance of dried raisins or caraway seeds; others of thin, flattened shreds of membrane, which, as they moved through the surrounding medium, bent from side to side with a waving motion, like that of an empty balloon of tissue-paper floating in the air." Some pure water was then added to the syrup, and the specimen again examined. "The whole of the corpuscles were seen changed: some had returned to their natural form, and were filling and becoming rounded, and thickened on their edges; others had already acquired a marked obicular character, and were so distended, that they appeared to bound, on coming in contact with other distended corpuscles, instead of gliding past them, as we observe in their natural condition." These phenomena were evidently the result of exosmosis and endosmosis, and are not a little interesting. If they shall be confirmed by other observers, they will serve to explain many points now involved in much obscurity.

viii. Mr. Wilkinson King has described a *Patella,* which appears to have been broken transversely, and is reunited by bone. The appearances are illustrated by a plate. He makes at the same time a number of desultory and not always very intelligible remarks upon the nature and treatment of such injuries, for which we must content ourselves with a simple reference to the paper.

IX. *A Case of Intestino-vesical Fistula, by* Mr. Hingeston. We have only room for an account of the dissection of the parts, of which a diagram and drawings are given :

"The colon—hypertrophied, singularly muscular, and in circumference about the size of a man's arm—was, together with a convolution of the ilium, and the appendix cœci, adherent to the fundus of the bladder. The natural course of its canal was impeded by a contraction or stricture, which commenced inferiorly in the rectum, about the forefinger's length from the anus, (just at the base of the triangle formed by the vesiculæ seminales,) and extended upwards for about two inches, barely admitting the entrance of the little finger. A section of the gut in this part resembled scirrhus; and the glands were, with the surrounding tissues, thickened. Immediately above this stricture the coats of the bowel were riddled with ulcerations and openings, leading into a channel which separated the bladder from the intestine. This channel was in fact a feculent abscess, situated beneath the reflected portion of the peritoneum, between the bladder and bowel. It was degenerate in structure, lined with a dark membrane, and filled with a muco-purulent excretion. It opened anteriorly, into the fundus of the bladder; above, into the colon; below, into the rectum; and posteriorly, through the colon into the ileum : so that there was a false passage, by which the natural course of the colon was diverted, and forced between the bladder and strictured part of the intestine down into the rectum below, and at the same place, by means of a fistulous opening, into the bladder in front. The orifice of this fistulous opening within the bladder was curtained by a fungous growth or thickening, which overhung it like a valve. Thus, exactly at this point of the feculent abscess, there was this strange deformation : the colon, the rectum, and the ileum, each, conjointly with the fecal abscess, possessed one common entrance into the bladder itself." (p. 406.)

It is an ungrateful task to find fault; but we feel it would be a dereliction of our duty did we neglect to stamp with our most decided disapprobation the vicious style in which this paper is composed. To give an instance: how can such expressions as the following be defended, either upon the score of taste or propriety? "So *silent* and insignificant was the starting *point* of the disease." "The false outlet through the bladder was lessened or *diverted.*" "A more alarming collapse, with a rapid pulse, clammy perspiration, obstinate hiccup, and all the other ominious and foreboding signs which usually *gather about the bed* in these perilous hours." "The *fistulous opening* into the bladder became finally *silent.*" We trust that Mr. Hingeston will henceforth avoid such injudicious misapplications of language; we are surprised they should be allowed by the editors to pass.

X. The last paper in this number is a communication on *Chorea*, from the pen of Dr. B. G. Babington. It is well and cleverly written, but contains nothing very novel. The author eulogizes Dr. Marshall Hall's explanation of the pathology of this disease, "that it is a morbid condition of the organ of emotion, which has its seat in the medulla oblongata, and is wholly independent either of the brain or the ganglionic system;" while he, at the same time, somewhat naïvely acknowledges "that it must be long before facts are accumulated, sufficiently numerous and decisive to establish or controvert this ingenious supposition." We do not think the cases recorded will avail much towards the establishment of the principle in question. It is clear that the only facts upon which reliance can be placed must be such as prove the existence of disease in the medulla oblongata, the supposed locality of this "organ of emotion;" but the post-mortem examinations before us present nothing of the kind, the nearest approach being the discovery, in one case, of old adhesions between the surfaces of the spinal arachnoid, "especially over the posterior part of the medulla." Disease of various parts of the brain and of the membranes of the spinal cord was indeed found in other cases, but these are manifestly inconclusive. Still the paper is interesting, and will well repay the trouble of perusing. We have room for little more than Dr. Babington's *definition* of chorea, which is somewhat novel:

"I should define chorea to be a disease characterized by irregular, uncontrollable contractions of the voluntary muscles, alternating with their atony, and occurring without pain..............I have used the word 'contractions,' and have included the atony of the muscles in this definition, because the movements in this disease appear to me to differ essentially from those of convulsions and epilepsy in this, that the stimulus, whatever be its nature, which excites either the whole or a portion of the voluntary muscles to involuntary action, is not more violent in degree than the normal stimulus of the will, or of the excito-motory system; so that movements almost incessant, indeed, but not exaggerated like spasms are the result............ The nerves, in their normal state, are always exercising a certain amount of influence over the muscles; so that where there is antagonism of forces it is only necessary to remove the one opponent in order to demonstrate that the other is in a state of activity. This being the healthy condition, we have a right to consider the diminution of this activity as a morbid state; for although, from the striking effect which a morbid exaltation of muscular force produces, spasm is more directly brought to the cognizance of our senses than atony, still the latter is no less really a morbid condition than the former. I venture, then, to express my belief, that while in true convulsions the muscles, after having been thrown into a spasmodic state, do only return to the normal condition; in chorea, on the contrary, a further diminution of nervous influence occurs, so that the muscles become, in all marked cases, entirely passive and inert in the intervals between their irregular and involuntary actions. This is manifest, from the manner in which the limbs drop from the position into which they have been thus thrown; in which the head, after being tossed to and fro, will fall passively on the shoulders; and from the incapability on the part of the patient to hold anything in his grasp." (p. 113.)

In regard to *treatment*, we do not find anything particularly new, excepting the notice of a mode of practice which has been lately adopted at St. Petersburg, with eminent success, in obstinate cases. The patient is placed in a bath as hot as he can bear it; kept there for half an hour; and, when thus thrown into the most profuse perspiration, is suddenly plunged into cold water. Among the innumerable tonic remedies which have been employed in this disease, Dr. Babington gives the preference to sulphate of zinc and the liquor arsenicalis. He also speaks highly of the shower-bath.

